# Association between victimization and perpetration of cyberhate: The moderating role of social dominance orientation

**DOI:** 10.1002/jad.12433

**Published:** 2024-10-22

**Authors:** Susett Naranjo‐Pou, Izabela Zych

**Affiliations:** ^1^ Department of Psychology University of Cordoba Cordoba Spain

**Keywords:** cyberhate, perpetration, social dominance, victimization

## Abstract

**Introduction:**

Expressions of cyberhate motivated by characteristics such as gender, ethnicity, and religious beliefs are now present and prevalent on social networks. Past research, both in online and offline contexts, has identified that, although there may be an overlap between victims and perpetrators of violence; this is not always the case. Nevertheless, the number of studies focused on variables that moderate the relation between victimization and perpetration is still low. The current study aims at analyzing the relation between cyberhate victimization and perpetration and the potential moderating role of social dominance on this relation.

**Method:**

During the 2019/2020 school year in Andalusia, Spain, a prospective longitudinal study was implemented. The study used a representative sample of 1498 adolescents enrolled in compulsory secondary education in the first wave (51.8% female; Mage = 13.58) and 1195 adolescents in the second wave (53.2% female; Mage = 14.04). Surveys administrated to adolescents were used for data collection.

**Results:**

The findings revealed a positive correlation between cyberhate victimization and perpetration. They supported the notion that social dominance not only predicted the perpetration of cyberhate several months later, but also its moderating effect on the relation between cyberhate victimization and perpetration. Cyberhate victims who reported higher levels of social dominance were more likely to become perpetrators several months later.

**Conclusion:**

Results suggest the need to implement preventive programs considering the influence of social dominance. These strategies could promote social equality and help to interrupt the cycle in which victims can become perpetrators of cyberhate.

## 1 INTRODUCTION

Cyberhate can be defined as any online expression that incite hatred, exclusion, and violence towards individuals or groups based on features such as gender, ethnicity, nationality, skin color, religious beliefs, sexual orientation, or other characteristics (Antidefamation League, 2016; Watanabe et al., 2018; Zhang & Luo, 2018). Although cyberhate is related to cyberbullying, the two terms are not synonymous (Kansok‐Dusche et al., 2022). The key difference lies in the target: while cyberbullying typically targets a specific individual or a small group, cyberhate extends its reach to entire communities, fostering violence among social groups (Blaya, 2019; Räsänen et al., 2016).

Cyberhate is present and prevalent in the daily online experiences of teenagers and young adults (Castaño‐Pulgarín et al., 2021; Hawdon et al., 2015; Näsi et al., 2015). Understanding cyberhate requires a thorough analysis of mechanisms behind victimization and perpetration, including their overlap and interaction. There is considerable evidence of overlap between victimization and perpetration in offline contexts, where victims can become perpetrators and vice versa (Broidy et al., 2006; Daday et al., 2005; Dobrin, 2001; Jennings et al., 2012; Unnever et al., 2016). Similar patterns have been observed in online contexts, though research in this area is still limited (Räsänen et al., 2016; Wachs & Wright, 2019). Furthermore, some individuals remain exclusively victims or perpetrators (Broidy et al., 2006; Jennings et al., 2012; Schreck et al., 2008; Zych et al., 2020), highlighting the need for more research to understand why some switch roles while others do not.

To address this gap, it is crucial to explore various factors that might influence transitions between victimization and perpetration. One such factor is social dominance orientation, a psychological construct that reflects an individual's preference for hierarchy and dominance over outgroups (Pratto et al., 1994). This study aimed to investigate a longitudinal relation between cyberhate victimization and perpetration among secondary education students in Spain. By examining the moderating role of social dominance orientation, this research seeks to understand the mechanisms behind the transition from victimization to perpetration and provide insights to inform policy and practice against cyberhate among adolescents.

### 1.1 Association between cyberhate victimization and perpetration

Research suggests that, while some people engage in cyberhate perpetration or victimization, it can sometimes be difficult to clearly classify individuals as perpetrators or victims. Räsänen et al. (2016) defined a perpetrator as someone who publishes or shares content containing online hate speech, and a victim as someone who feels belittled, hated, or attacked by such material. In a study involving a sample of young Finns, they found that the likelihood of victimization is significantly higher when the participant has previously produced hate material. This association between cyberhate victimization and perpetration has also been found in other studies (Wachs & Wright, 2019; Wright & Li, 2013).

There are several theoretical explanations of the victim‐perpetrator overlap. According to the social learning theory (Bandura & Walters, 1977), individuals can internalize behavioral patterns through the observation of others, subsequently perceiving the utility of observed behavior which can be repeated. In this sense, victims of cyberhate might become perpetrators as they have learned aggressive behaviors through their experiences.

Another perspective comes from routine activity theory (Cohen & Felson, 1979), which refers that victimization is more likely when a motivated offender encounters a potential victim in absence of a capable guardian. This theory emphasizes the influence that environmental factors have on increasing the likelihood of both victimization and aggression. Osgood et al. (1996) highlighted that delinquency is driven not by time spent in hostile environments, but by the lack of adult supervision, a factor particularly relevant to cyberspace, where interactions can occur without such oversight (Marcum, 2011). Additionally, Finkelhor and Asdigian (1996) expanded on this theory by introducing the concept of “target antagonism,” which suggests that victims are not merely passive recipients of aggression, but their characteristics or behaviors can make them more vulnerable to further attacks, potentially escalating cycles of hostility. As Black (1984) notes, violence begets violence; thus, a grievance pursued aggressively can lead to aggression in return. In the context of cyberhate, victims could retaliate against their attackers, perpetuating the cycle of hate.

This cycle is further reinforced by the adoption of maladaptive coping strategies, such as seeking revenge, that may also contribute to victims of cyberhate becoming perpetrators (Wachs et al., 2020). As stated by Singer (1981), individuals who have committed acts of aggression may become susceptible to retaliatory actions from their prior victims, within the context of the violence subculture. Thus, there are reasons to believe that cyberhate victimization could increase the risk of cyberhate perpetration, but the mechanisms behind this relation still need to be clarified. Wachs and Wright (2019), drew attention to the need for longitudinal studies to understand the nature of victimization and perpetration of cyberhate.

### 1.2 Social dominance orientation as a moderator of the association between victimization and perpetration of cyberhate

Social dominance orientation refers to a psychological mechanism aimed at the establishment and maintenance of social hierarchies and unequal relationships (Pratto et al., 1994). Essentially, it signifies a propensity to endorse the superiority of one's ingroup or affiliative group over outgroups, thereby perpetuating a power imbalance. In fact, Watanabe et al. (2018) stated that in the online realm, many Internet users tend to employ hateful speech when interacting with people who do not share similar opinions, beliefs, and background. This might be caused by the perception of the superiority of one's ingroup over outgroups.

Adolescents who are still developing their identities can be particularly susceptible to the influences of social dominance when they navigate in online spaces. The anonymity and disinhibition effects of digital environments (Wachs & Wright, 2018), along with adolescents' inclination to violate social norms, the importance of peer acceptance and the ongoing development of emotional regulation (Ghisleri & Samada, 2022), may contribute to the expression of social dominance in the online realm. An adolescent with high social dominance may find in the online realm a space to support social inequality and spread prejudice, especially if such behavior is reinforced by social approval, such as receiving likes or reposts from peers.

Although research on the relation between social dominance and cyberhate is limited, a study by Castellanos et al. (2024) with adolescents demonstrated that social dominance is positively associated with hate speech perpetration both offline and online. These authors also found that social dominance has indirect effects on hate speech through low levels of empathy and high levels of moral disengagement. Furthermore, social dominance has been empirically linked to other variables related to prejudice, such as authoritarianism (Etchezahar, 2017), gender stereotypes (Castillo‐Mayén, 2010), homophobic name‐calling perpetration (Valido et al., 2022), and intra‐ethnic bullying/victimization.

Wachs et al. (2024) investigated four profiles of bystander of racist hate speech among adolescents: passive, defender, revenger, and contributor. They concluded that bystanders with high social dominance orientation were more inclined to ignore hate speech incidents or participate actively in them. Thus, these students were more likely to be classified as revengers or contributors. On the other hand, students with low social dominance were more inclined to oppose hate speech and even support the targeted group.

These findings support the idea that social dominance orientation could represent a steppingstone that supports and fuels hate speech not only offline but also in online contexts. A central premise among individuals and groups promoting hate speech is that any behavior aimed at defending the power and status of a dominant social group is “justified” (Vaught, 2012). Indeed, social dominance may serve as a potential moderating variable in the association between cyberhate victimization and perpetration. High level of social dominance implies viewing inequality as a natural element of interpersonal and intergroup dynamics. Therefore, if victims have this belief framework, it could be conceivable that they seek to carry from a position of subordination to one of authority. In this case, victims of cyberhate could replicate the perpetrator model and behave accordingly to exert greater power.

### 1.3 The current study

At both theoretical and empirical fronts, the number of studies on cyberhate is still low and there are pressing gaps in knowledge that need to be addressed promptly (Bauman et al., 2021; Blaya, 2019; Castaño‐Pulgarín et al., 2021; Chetty & Alathur, 2018; Hawdon et al., 2015; Tareen et al., 2021). The present study aimed to analyze the association between cyberhate victimization and perpetration and to explore the moderating effect of social dominance orientation on the link between cyberhate victimization and subsequent perpetration among Andalusian adolescents. There is a possibility that victims may become perpetrators several months later if they score high on social dominance, as they may hold the belief that ceasing to be dominated always implies dominating others. Conversely, victims who score low on social dominance may not be motivated to dominate others and may employ alternative strategies to end victimization.

Thus, it was hypothesized that i. cyberhate victimization is related to and predicts later cyberhate perpetration, ii. social dominance orientation predicts cyberhate perpetration a few months later, and iii. the association between cyberhate victimization and subsequent cyberhate perpetration is stronger at higher levels of social dominance orientation.

## 2 MATERIAL AND METHODS

### 2.1 Participants

The sample was randomly selected from the population of 399.553 compulsory secondary education students during the 2019/2020 school year in Andalusia, Spain. To ensure representativeness, a multi‐stage stratified random sampling approach was employed, considering the proportional distribution of students across each of the eight Andalusian provinces: Almeria, Cadiz, Cordoba, Granada, Huelva, Jaen, Malaga, and Seville. Schools were considered as clusters, and it was estimated that selecting one classroom from each grade (1–4) in each school would give at least 80 students in each school (20 per classroom). With these considerations, 21 schools were randomly selected to be included in this study.

In the initial wave (W1) of data collection, data from a representative sample of 1,498 students was gathered between October and December 2020. Given the average class size in secondary education in Andalusia and that data were collected from 83 class groups, the initial estimated total target sample size was of around 1660 students. Of these, data from 1,498 students were obtained, resulting in an estimated overall response rate of around 90%.

The sample consisted of 51.8% girls and 48.2% boys, with ages ranging from 11 to 17 years (M = 13.58; SD = 1.33). The distribution of participants across grades was as follows: first grade (27.6%), second grade (23.2%), third grade (25.7%), and fourth grade (23.5%).

In the second wave (W2), 1,195 students were followed up and data was collected between May and June 2021. The sample consisted of 53.2% girls and 46.8% boys, between the ages of 11 and 17 years (M = 14.04; SD = 1.35). The distribution across grades in W2 was as follows: first grade (27.7%), second grade (23.3%), third grade (26.2%), and fourth grade (22.8%). Thus, 79.77% of the participants from W1 remained in the study in W2 with a high retention rate between the two waves of data collection. Students who completed both waves and those who did not were compared using Student‐t‐test across the main study variables. No significant W1 differences were observed for cyberhate victimization (*p* > 0.05) and social dominance orientation (*p* > 0.05). However, a small but statistically significant difference was found for cyberhate perpetration (*t* (365.455) = 2.725, *p* = 0.003, Cohen's *d* = 0.357), with higher scores observed among students who did not complete both waves. Given the small effect size and that this study focused on relations among variables, it is unlikely that this difference would significantly impact the overall findings.

### 2.2 Measures

The Cordoba Cyberhate Questionnaire (CCQ; Zych et al., 2021) was used to measure victimization and perpetration of cyberhate, each with 10 items. The participants responded to each item using a 5‐point Likert scale ranging from 1 (*no*) to 5 (*yes, more than once a week*). Items measured online expression of hate towards minority groups based on their religion, culture, sexual orientation, skin color, gender, and other characteristics experienced within one school year (the past school year in W1 and the current school year in W2). The perpetration subscale aimed to identify expressions of cyberhate, while the victimization subscale assessed participants' experiences of receiving cyberhate (e.g., ‘I have expressed hatred towards immigrants, some religions, or people of a certain skin color online.’). On the other hand, as part of the victimization subscale, participants were asked about the receipt of cyberhate (e.g., ‘Someone has expressed hatred towards my minority group for being immigrants, for our religion, or for the color of our skin online.’). See all the items in Supporting Information S1: Appendix S1.

A Confirmatory Factor Analysis (CFA) was conducted using the robust method. The fit indices indicated a good model fit: Satorra‐Bentler scaled chi‐square = 769.98, df = 402, *p* < 0.001; NFI = 0.96; NNFI = 0.98; CFI = 0.98; IFI = 0.98; RMSEA = 0.038 (90% CI: 0.034, 0.042). Both subscales had an excellent reliability in the current sample in W1 (*α* = 0.90; *Ω* = 0.90 for perpetration and *α* = 0.96; *Ω* = 0.96 for victimization) and in W2 (*α* = 0.96; *Ω* = 0.96 for perpetration and *α* = 0.97; *Ω* = 0.97 for victimization). These results suggest that the factorial structure of the scale is valid and reliable, demonstrating good fit to the observed data.

Abbreviated version of the Social Dominance Orientation Scale was used to measure social dominance using a reduced scale from the attitudinal orientation questionnaire towards social dominance proposed by Pratto et al. (1994). Three items were included that indicate approval of inequality between groups (e.g., ‘It is okay that some groups have more opportunities in life than others’). See all the items in Supporting Information S1: Appendix S2. The response scale used a 7‐point Likert format ranging from 1 (*strongly disagree*) to 7 (*strongly agree*). In both administrations, the scale demonstrated good internal consistency (W1: *α* = 0.81; *Ω* = 0.82 and W2: *α* = 0.84; *Ω* = 0.85).

### 2.3 Design and procedure

A prospective longitudinal design was implemented for the current study, using a random selection of schools from all the Andalusian provinces. The majority of questionnaires (92.2% of schools) were administered in a paper‐and‐pencil survey within a quiet classroom setting during regular school hours. However, two schools (7.1%) opted for participating online due to logistical constraints related to the COVID‐19 pandemic. There was no statistically significant difference between the paper‐and‐pencil and online administered responses in cyberhate perpetration and victimization or social dominance orientation (*p* > 0.05).

At the beginning of the 2020/2021 school year, the head teachers were approached and provided with an explanation of the study objectives. Upon obtaining approval from the school boards, parental consent was obtained for each participant. Participants were informed of the anonymous and confidential nature of the study and that their participation was voluntary. Questionnaires were filled in during regular classroom hours in a quiet environment, taking approximately 45 min. The researchers supervised the students during this process, collecting the questionnaires without any teacher intervention, thus ensuring equal environments across all schools and safeguarding student privacy. Teachers did not have access to individual questionnaires or student data. The questionnaires from W1 and W2 were linked using an anonymous code. This study adhered to national and international ethical standards and has received approval from the Ethics Committee of the University of Cordoba, Spain.

### 2.4 Data analysis

The reliability of the research instruments was assessed by calculating the Cronbach's alpha coefficients and McDonald's omegas, employing the FACTOR 10 software (Lorenzo‐Seva & Ferrando, 2015). To examine the direct bivariate relations between cyberhate perpetration, cyberhate victimization, and social dominance at both W1 and W2, Pearson correlations were calculated using the statistical package SPSS (version 25).

To test the study's hypotheses, a regression‐based moderated model was run using the Process Macro (Hayes, 2013; version 4.2, model 1), utilizing a bootstrap sampling of 5000 and a 95% confidence interval. All variables were mean centered before creating the interaction term. The independent variable was W1 cyberhate victimization (X), with W1 social dominance orientation (W) as the moderating variable, and W2 cyberhate perpetration as the dependent variable (Y). All analyses were controlled for participants' age, gender and W1 cyberhate perpetration. Standardized regression coefficients were obtained by standardizing the variables. Effect sizes for social dominance orientation levels were determined using Cohen's *f²*, with values of *f²* ≥ 0.03, *f²* ≥ 0.04, and *f²* ≥ 0.09 representing small, medium, and large effect sizes, respectively.

## 3 RESULTS

### 3.1 Association between cyberhate and social dominance orientation

For cyberhate perpetration in W1, the mean was *M* = 1.20 (SD = 0.36). For victimization, the mean was *M* = 1.29 (SD = 0.62) indicating that most respondents reported low levels of cyberhate perpetration and victimization. In W2, perpetration increased slightly with a mean of *M* = 1.85 (SD = 0.95), while victimization also increased to *M* = 1.31 (SD = 0.66). Bivariate correlations between cyberhate perpetration, cyberhate victimization, and social dominance orientation at first wave (W1) and second wave (W2) are shown in Table 1.

**Table 1:** Correlations between cyberhate perpetration, cyberhate victimization and social dominance orientation at W1 and W2.

	1	2	3	4	5
1. Cyberhate perpetration W1	1				
2. Cyberhate victimization W1	0.27*	1			
3. Social dominance W1	0.29*	0.03	1		
4. Cyberhate perpetration W2	0.41*	0.16*	0.26*	1	
5. Cyberhate victimization W2	0.11*	0.63*	0.05	0.29*	1
6. Social dominance W2	0.27*	<0.01	0.44*	0.29*	0.04

* *p* < 0.01

Higher levels of cyberhate victimization and social dominance orientation were related to more cyberhate perpetration, both at W1 and W2. There was also a cross‐sectional and longitudinal relation between cyberhate victimization and perpetration. Furthermore, each measured variable at W1 was positively related to the same variable at W2. There was no evidence of the relation between cyberhate victimization and social dominance orientation.

### 3.2 Cyberhate victimization as a predictor of cyberhate perpetration moderated by social dominance

Longitudinal predictors of cyberhate perpetration are shown in Table 2. The model proved to be statistically significant (*F*_(6,0000)_ = 46.09; *p* < 0.001; *R²* = 0.20). Specifically, cyberhate victimization at the onset of the school year emerged as a salient predictor of increased cyberhate perpetration by the end of the school year (*β* = 0.05, *p* = 0.001). Higher levels of individual's social dominance orientation at the initial time point were positively associated with cyberhate perpetration at the later time point (*β* = 0.04, *p* < 0.001). Similarly, cyberhate perpetration at the beginning of the school year was associated with subsequent acts of cyberhate by the end of the school year (*β* = 0.35, *p* < 0.001). However, age and sex were not significant predictors of perpetration of cyberhate.

**Table 2:** Cyberhate victimization and social dominance orientation as longitudinal predictors of cyberhate perpetration.

	β [IC 95%]	SE	*t*	*p*
Constant	0.65 [0.45–0.85]	0.10	6.47	<0.001
Cyberhate victimization W1	0.05 [0.02–0.08]	0.02	3.22	0.001
Social dominance W1	0.04 [0.03–0.06]	0.01	5.62	<0.001
Interaction	0.02 [0.00–0.04]	0.01	2.03	0.04
Cyberhate perpetration W1	0.35 [0.28–0.41]	0.03	10.80	<0.001
Age	0.01 [−0.01–0.02]	0.01	1.12	0.26
Sex (male)	0.02 [−0.02–0.06]	0.02	1.08	0.28

*Note*: Interaction refers interaction between cyberhate victimization W1 and social dominance W1.

**Figure 1:**
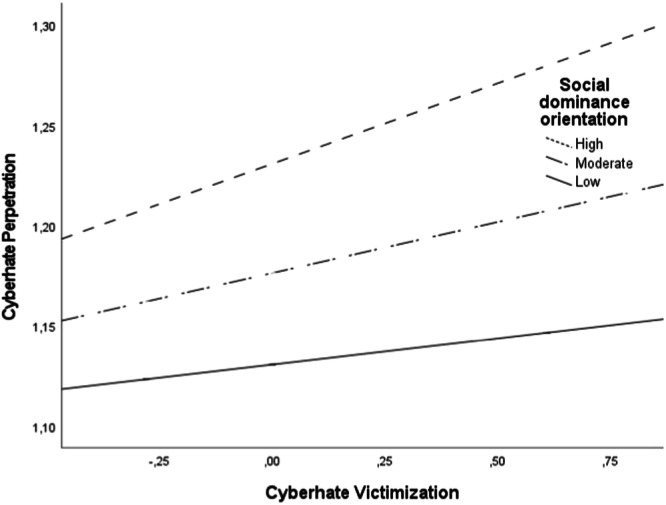
Graphical representation of the moderation of social dominance orientation on the association between cyberhate victimization and perpetration. Age, gender and W1 cyberhate perpetration were controlled for in the analysis.

A significant moderation effect was found between victimization of cyberhate and social dominance orientation at the initial time points, when predicting perpetration of cyberhate by the end of the school year (*β* = 0.02, *p* = 0.04). The analysis also unveiled a nuanced relationship between cyberhate victimization, social dominance orientation, and later cyberhate perpetration. Victims of cyberhate at the beginning of the school year (W1) were more likely to engage in cyberhate perpetration a few months later (W2) if they initially reported higher levels of social dominance (*β* = 0.08 [0.04–0.13], *p* < 0.001, *SE* = 0.02 at +1 SD). This association remained significant but weaker for individuals who reported moderate levels of social dominance (*β* = 0.05 [0.02–0.08], *p* = 0.001; *SE* = 0.02). Conversely, for those who reported lower levels of social dominance orientation at W1, no significant link was found between being a victim of cyberhate and engaging in cyberhate perpetration at W2 (*β* = .03 [−0.01–0.06], *p* = 0.16; *SE* = 0.02 at −1 SD) (Figure 1).

The data further elucidate the escalating role of social dominance orientation as a moderating variable. As the level of social dominance orientation increased, its moderating effect also intensified, starting with a Cohen's *f* ^2^ = 0.03 and progressing to a Cohen's *f* ^*2*^ = 0.16. This indicates that social dominance orientation plays a relevant role in moderating the intensity of the relationship between cyberhate victimization and subsequent perpetration. In summary, the findings suggest that an increase in the level of social dominance orientation amplifies the likelihood that individuals who have been victims of cyberhate will transition into roles of cyberhate perpetration several months later.

## 4 DISCUSSION

The primary aims of this research were twofold: first, to analyze the relationship between cyberhate victimization and cyberhate perpetration; and second, to explore the moderating effect of social dominance orientation on the link between cyberhate victimization and ensuing perpetration. Consistent with our first hypothesis, a positive correlation was observed between cyberhate victimization and cyberhate perpetration at both time points. Notably, we found that cyberhate victimization at the initial time point could predict cyberhate perpetration at the later time point. These findings are congruent with prior research demonstrating an association between victimization and aggressive behavior, both in offline contexts (Jennings et al., 2012; Schreck et al., 2008) and in the cyber realm (Wachs & Wright, 2019; Wachs, Wright & Vazsonyi, 2019; Wright & Li, 2013). The underlying mechanisms that may account for this relationship encompass various factors such as social learning, environmental influences, high‐risk lifestyles, and maladaptive coping strategies, including retaliatory actions (Jennings et al., 2012; Schreck et al., 2008; Singer, 1981; Wachs et al., 2020).

Pertaining to our second hypothesis, the data substantiated that social dominance orientation was a significant predictor of cyberhate perpetration several months later. This finding is consistent with research by Castellanos et al. (2024), who demonstrated that social dominance was positively associated with perpetration of cyberhate. It also aligns with research conducted by Wachs et al. (2024), which showed that bystanders with high social dominance were more likely to actively participate in hate speech events. Watanabe et al. (2018), argued that an imbalanced perception of power serves as a catalyst for cyberhate manifestations.

Regarding our third hypothesis, positing that social dominance orientation moderates the relation between cyberhate victimization and subsequent perpetration, received empirical support. The analysis revealed a nuanced relationship: victims who reported higher levels of social dominance orientation at the beginning of the school year were more likely to engage in perpetration by the end of the school year. Conversely, those who reported lower levels of social dominance orientation were less likely to perpetrate cyberhate by the end of the year.

The concept of target antagonism within the framework of routine activity theory, may provide valuable insights into interpreting this finding (Finkelhor & Asdigian, 1996). Rather than viewing the victim as a passive subject, this perspective emphasizes how certain victim characteristics can make them vulnerable to the anger or resentment of the perpetrator. In the context of cyberhate, some victims, instead of remaining passive recipients of aggression or adopting assertive coping strategies, may respond with hostility as a form of retaliation. Social dominance orientation may amplify this response by reinforcing beliefs in hierarchical social structures where power imbalances are not only accepted but pursued. Victims with high social dominance might see retaliatory aggression to assert dominance and shift from a position of subordination to one of control, thereby transforming into perpetrators of cyberhate.

Given these insights, targeted preventive measures should aim to reduce social dominance orientation among adolescents, as a means of preventing victims of cyberhate from becoming perpetrators. Educational institutions should implement programs that foster empathy, respect for diversity, and critical thinking about power dynamics. For instance, incorporating curriculum content that addresses the dangers of social hierarchies and promotes equality could be effective. Educators should be trained to recognize signs of social dominance orientation and address these attitudes through both classroom instruction and individual counseling.

However, due to the relatively small effect sizes observed for social dominance in the relation between victimization and perpetration of cyberhate, results should be interpreted with caution. Interventions should not focus exclusively on social dominance and other variables, such as online toxic disinhibition (Wachs & Wright, 2019), peer rejection (Wright & Li, 2013), low empathy and moral disengagement (Castellanos et al., 2024), also play critical roles and should be addressed within comprehensive intervention strategies. Other underlying mechanisms should be urgently studied and included in interventions against cyberhate.

In general, Spain exhibits notable ethnic and cultural diversity. According to provisional data from the *Instituto Nacional de Estadística* (Spain's National Institute of Statistics, 2024) the foreign population as of July 2024 experienced a significant increase of 4.63% compared to the same period the previous year. Specifically, in January 2020, there were 222.223 foreign individuals aged 10 to 14 years (9.68% of the population), which rose to 285.397 by January 2023 (12.72% of the population). These statistics highlight the growing ethnic and cultural diversity within Spain's educational institutions.

Allport, (1954) intergroup contact theory suggests that under certain conditions, contact between groups can foster acceptance and reduce prejudice. This theory may help explain the relatively low levels of cyberhate among Spanish students in this study, as classrooms provide a heterogeneous environment where students from different ethnicities and cultural backgrounds frequently interact. Cultural diversity in Spain is key for designing effective interventions, and teachers play a critical role in fostering the conditions to reduce prejudice. Allport highlights four factors for successful prejudice reduction: equal group status, common goals, intergroup cooperation, and authority support.

School and community organizations could design and evaluate comprehensive interventions to tackle cyberhate and its ramifications. Such initiatives would serve dual purposes: firstly, they would prevent the normalization of this detrimental phenomenon; secondly, they would facilitate the development of adaptive coping strategies to confront and mitigate its impact. These strategies should be comprehensive and ecological and could include assertiveness training, promoting social and emotional competencies (Marín‐López & Zych, 2024), positive school climate (Casas et al., 2013; Llorent et al., 2021) and garnering close support to seeking distal advice and employing technical means to cope, as suggested by prior research (Gámez‐Guadix et al., 2020; Wachs et al., 2020).

Beyond coping, it is equally imperative for adolescents to understand their civic duties and rights. This encompasses an appreciation for the tenets of social responsibility and respect for all individuals, both in the digital and physical worlds. Consequently, establishing guidelines that require schools to integrate digital citizenship education into their curriculum may be essential. This education should include lessons on the cybersecurity, relevant legal frameworks, and the psychosocial ramifications of violating these norms are of the utmost importance.

In this context, Wachs and Wright (2019) emphasized the pivotal role that educators and family members play in guiding, instructing, and supporting adolescents as they navigate the complexities of digital interactions. In fact, Wright et al. (2021) identified that instructive parental mediation and family support showed a positive impact on the adolescents ability to cope healthily with expressions of cyberhate. Such a multi‐faceted approach can serve as a robust framework for fostering responsible and respectful online behavior among adolescents.

### 4.1 Strengths, limitations, and future research

The present study boasts several notable strengths, including a representative sample of Andalusian adolescents, and the use of a longitudinal design that allows for the temporal examination of variables. The low attrition rate during the second evaluation period further enhances the validity of our findings, as retention rates ≥70% are rated as high quality according to the Cambridge Quality Checklist (Murray et al., 2009). However, the study is not without limitations. The data is solely reliant on self‐reported measures, which introduces the possibility of response bias and future research might benefit from supplementing this approach with other qualitative and quantitative instruments to triangulate data and provide a more comprehensive understanding of the studied phenomena.

In addition, although a small but significant difference was observed in cyberhate perpetration between the participants who completed both waves and those who did not, the effect size was modest, indicating limited practical impact. Nevertheless, we acknowledge that this difference could introduce some bias, particularly in the interpretation of perpetration patterns. Future research should consider implementing more robust retention strategies to further mitigate the potential influence of attrition.

While social dominance orientation was found to be a significant moderator in the relation between victimization and perpetration of cyberhate, the observed effect sizes were modest, suggesting that caution is necessary when interpreting these findings. It is crucial to explore other potential moderating or mediating variables such as peer influence in both online and offline contexts, social emotional competences, parenting styles, school climate and coping strategies, all of which may play critical roles in shaping the victim‐perpetrator dynamic.

Future research should aim to undertake further longitudinal studies to explore the effects of these and other variables. For instance, examining the role of witnesses to cyberhate could be pivotal; bystanders often possess significant untapped potential to intervene and counteract online hate speech (Wachs et al., 2024). Identifying both risk and protective factors related to cyberhate can also provide valuable insights. Developing reliable and valid measures for this purpose, as suggested by Wachs and Wright (2019), can aid in the identification of vulnerable populations, thereby facilitating the creation of targeted prevention and intervention programs.

## 5 CONCLUSIONS

The growing concern about cyberhate, though relatively recent, is already resulting in research evidence about its considerable adverse impacts (Castaño‐Pulgarín et al., 2021; Kansok‐Dusche et al., 2022; Tareen et al., 2021). This troubling trend poses a serious challenge to national and international initiatives aimed at fostering inclusion, equity, and combating inequality (Canadian Centre for Child Protection, 2021; Gobierno de España, 2018; World Health Organization, 2016). In light of these concerns, it is our aspiration that the present study offers valuable insights into the intersecting roles of victims and perpetrators in the realm of cyberhate. We provided evidence that social dominance is a mechanism that may help explain why some cyberhate victims could become perpetrators several months later. These findings, alongside other relevant factors, could contribute to the development of programs aimed at preventing cyberhate. Given the burgeoning scale of the issue, there exists a social imperative to make cyberspace a more secure environment for all users. Raising awareness, providing education, and establishing legitimate avenues for adolescents to seek assistance and support are crucial steps in this direction.

## Supporting information

Supporting information.

## Data Availability

The data that support the findings of this study are available from the corresponding author upon reasonable request.
