# Supplementary material for: Association between victimization and perpetration of cyberhate: The moderating role of social dominance orientation
Source: J Adolesc. 2024 Oct 22;97(2):479–88. doi: 10.1002/jad.12433 (PMC11791727; doi:10.1002/jad.12433)
Supplement: Supplementary file 1 — Supporting information. [file JAD-97-479-s001.docx]

**Appendix 1**

**The Cordoba Cyberhate Questionnaire**

Students were asked about perpetration, victimization, and witnessing of cyberhate in the last year. Questions were focused on hate towards minorities. Minorities are people who have a culture, religion, sexual orientation, skin color, socioeconomic status, educational needs, disability, etc. that are different from most people in Spain.

| 1 | 2 | 3 | 4 | 5 |
| --- | --- | --- | --- | --- |
| No/No | Sí, una o dos veces /Yes, once or twice | Sí, una o dos veces al mes/ Yes, once or twice a month | Sí, alrededor de una vez a la semana/ Yes, about once a week | Sí, más de una vez a la semana/ Yes, more than once a week |

| **CYBERHATE PERPETRATION** |  |
| --- | --- |
| 1. He expresado odio hacia inmigrantes, algunas religiones o gente de cierto color de piel por internet / I have expressed hatred towards immigrants, some religions or people of a certain skin color online |  |
| 2. He expresado odio hacia ciertas orientaciones sexuales por internet / I have expressed hatred towards certain sexual orientations online |  |
| 3. He expresado odio hacia cierto género por internet / I have expressed hatred towards a certain gender on the internet |  |
| 4. He mandado mensajes a foros o redes sociales que expresan odio hacia minorías / I have sent messages to forums or social networks that express hatred towards minorities |  |
| 5. He hecho fotos o vídeos mostrando odio hacia minorías por internet / I have taken photos or videos showing hatred towards minorities online |  |
| 6. He compartido fotos o vídeos con odio hacia minorías por internet / I have shared photos or videos with hatred towards minorities online |  |
| 7. Me he relacionado con grupos que odian a ciertas minorías por internet / I have been associated with groups that hate certain minorities online |  |
| 8. Procuro que los demás se den cuenta a través del internet de que algunas minorías son odiosas / I try to make others realize through the internet that some minorities are hateful |  |
| 9. He incitado a la gente por internet a que use la violencia física contra ciertas minorías / I have incited people online to use physical violence against certain minorities |  |
| 10. Hago saber a la gente por internet que algunas minorías deberían desaparecer / I let people know online that some minorities should disappear |  |

| **CYBERHATE VICTIMIZATION** |  |
| --- | --- |
| 1. Alguien ha expresado odio hacia mi grupo de minoría por ser inmigrantes, por nuestra religión o por el color de nuestra piel por internet / Someone has expressed hatred towards my minority group for being immigrants, for our religion or for the color of our skin online |  |
| 2. Alguien ha expresado odio hacia mi grupo de minoría por nuestra orientación sexual por internet / Someone has expressed hatred towards my minority group for our sexual orientation online |  |
| 3. Alguien ha expresado odio hacia mi género por internet / Someone has expressed hatred towards my gender online |  |
| 4. Alguien he mandado mensajes a foros o redes sociales que expresan odio hacia mi minoría / Someone has sent messages to forums or social networks expressing hatred towards my minority |  |
| 5. Alguien ha hecho fotos o vídeos mostrando odio hacia mi minoría por internet / Someone has taken photos or videos showing hatred towards my minority on the internet |  |
| 6. Alguien ha compartido fotos o vídeos con odio hacia mi minoría por internet / Someone has shared photos or videos with hatred towards my minority online |  |
| 7. Mi minoría ha sido víctima de grupos que odian a ciertas minorías por internet / My minority has been the victim of groups that hate certain minorities online |  |
| 8. Alguien ha procurado que los demás se den cuenta a través del internet de que mi minoría es odiosa / Someone has tried to make others realize through the internet that my minority is hateful |  |
| 9. Alguien ha incitado a la gente por internet a que use la violencia física contra mi minoría / Someone has incited people online to use physical violence against my minority |  |
| 10. Alguien ha hecho saber a la gente por internet que mi minoría debería desaparecer / Someone has let people know online that my minority should disappear |  |

**Appendix 2**

**Social Dominance**

Questions about social dominance thoughts in a 3-items questionnaire.

| 1 | 2 | 3 | 4 | 5 | 6 | 7 |
| --- | --- | --- | --- | --- | --- | --- |
| Fuerte desacuerdo/ Strongly disagree | Desacuerdo/disagree | Algo desacuerdo/ Somewhat disagree | Neutro/ Neutral | Algo de acuerdo/ Somewhat agree | De acuerdo/ Agree | Fuerte acuerdo/ Strongly agree |

| **Dominacia Socia**l |  |
| --- | --- |
| Algunos grupos de personas son simplemente inferiores a otros grupos / Some groups of people are simply inferior to other groups |  |
| Está bien que algunos grupos tengan más oportunidades en la vida que otros / It is okay that some groups have more opportunities in life than others |  |
| Es bueno que ciertos grupos queden por encima y otros por debajo / It is good that certain groups are above and others below |  |
